# Characterization of the resistance class 1 integrons in *Staphylococcus aureus* isolates from milk of lactating dairy cattle in Northwestern China

**DOI:** 10.1186/s12917-018-1376-5

**Published:** 2018-02-27

**Authors:** Longping Li, Xin Zhao

**Affiliations:** 1 College of Animal Science and Technology, Northwest A&F University, YangLing, Shaanxi People’s Republic of China; 2 Life Science Research Center, Yulin University, Yulin, 719000 People’s Republic of China; 3 Shaanxi Provincial Engineering and Technology Research Center of Cashmere Goat, Yulin University, Yulin, 719000 People’s Republic of China; 4 Department of Animal Science, McGill University, 21,111 Lakeshore, Ste. Anne de Bellevue, Quebec, H9X 3V9 Canada

**Keywords:** Class 1 integron, Gene cassettes, Antibiotic resistance, Bovine milk, *Staphylococcus aureus*

## Abstract

**Background:**

Integrons are mobile DNA elements and they have an important role in acquisition and dissemination of antimicrobial resistance genes. However, there are limited data available on integrons of *Staphylococcus aureus* (*S. aureus*) from bovine mastitis, especially from Chinese dairy cows. To address this knowledge gap, bovine mastitis-inducing *S. aureus* isolates were investigated for the presence of integrons as well as characterization of gene cassettes. Integrons were detected using PCR reactions and then further characterized by a restriction fragment-length polymorphism analysis and amplicon sequencing.

**Results:**

All 121 *S. aureus* isolates carried the class 1 integrase gene *intI*1, with no *intI*2 and *intI*3 genes detected. One hundred and three isolates were positive for the presence of 12 resistance genes, either alone or in combination with other gene cassettes. These resistance genes encoded resistance to trimethoprim (*dhfrV, dfrA1, dfrA12*), aminoglycosides (*aadA1, aadA5, aadA4, aadA24, aacA4, aadA2, aadB*), chloramphenicol (*cmlA6*) and quaternary ammonium compound (*qacH*) and were organized into 11 different gene cassettes arrangements (A-K). The gene cassette arrays *dfrA1-aadA1* (D, 44.6%), *aadA2* (K, 31.4%), *dfrA12-orfX2-aadA2* (G, 27.3%) and *aadA1* (A, 25.6%) were most prevalent. Furthermore, 74 isolates contained combinations of 2 to 4 gene cassette arrays. Finally, all of the integron/cassettes-positive isolates were resistant to aminoglycoside antibiotics.

**Conclusions:**

This is the first study on the integrons and gene cassette arrays in *S. aureus* isolates from milk of mastitic cows from Northwestern China and provide the evidence for class 1 integron as possible antibiotic resistance determinants on dairy farms.

## Background

*Staphylococcus aureus (S. aureus)* induced bovine mastitis causes heavy losses for the dairy industry worldwide. In addition, acquisition and spread of antibiotic resistance among staphylococcal strains is a major concern in treatment of staphylococcal infections in humans and animals. The evolution of multidrug resistance is relatively fast due to horizontal or lateral gene transfer, which is influenced by a wide range of mobile genetic elements [1]. In particular, integrons comprise a substantial proportion of these elements and are often found in plasmids and/or transposons that enhance spread of resistance genes [2].

Integrons are mobile DNA elements that are composed of three DNA segments, including a 5′ conserved segment (5’CS), an internal variable region with one or more resistance gene cassettes of different lengths and sequences, and a 3′ conserved segment (3’CS). The 5’CS encodes a recombinase (integrase) which allows for site-specific insertion of resistance-gene cassettes between two highly conserved adjacent nucleotide sequences (5’CS and 3’CS) and a promoter that ensures expression of the integrated gene cassettes. The 3’CS contains a quaternary ammonium compound resistance gene *qacΔE*-1, the sulfonamide resistance gene *sul*1 and an open reading frame of an unknown function [3]. At present, at last 5 classes of mobile integrons have been distinguished by their highly conserved class-specific integrase genes [3] and gene cassettes are widely disseminated in diverse bacteria [2]. Mobile integrons have been a major driver in the spread of antibiotic resistance and ongoing use of antibiotics has swelled their numbers in recent years [2].

The role of class 1 integrons is well known in the spread of antibiotic resistance genes in gram-negative bacteria [4, 5]. A previous study has also shown that gram-positive bacteria were a major reservoir of class 1 antibiotic resistance integrons in poultry litter [6]. Furthermore, nosocomial infection caused by resistance class 1 integron-carrying *S. aureus* [7] or methicillin-resistant *S. aureus* [8, 9] were also reported in Southern China. However, investigation of integrons in bovine milk-associated *S. aureus* is still lacking. Therefore, the aims of this work were (i) to determine the class 1 integron-associated genes in *S. aureus* isolates obtained from dairy farms in Shaanxi province in Northwestern China and (ii) to amplify and characterize their gene cassette contents and antibiotic resistance gene cassette arrays by sequencing of *S. aureus* isolates.

## Methods

### Bacterial strains and susceptibility testing

Milk samples were collected from 4 dairy farms in three cities (BaoJi, XiAn and XianYang) in the Shaanxi Province. These 4 farms had 24 to 68 lactating cows and samples were collected between September 2011 and October 2012. Milk samples were taken from cows with clinical mastitis. Cows were selected by veterinarians based on clinical mastitis which was manifested with decreased milk production, color change of the milk and inflammation of udders. Milk samples were collected after cleaning the teats, discarding a few streams of milk and scrubbing the teat ends with cotton balls moistened with 70% alcohol. In all, 121 *S. aureus* isolates were isolated from 214 individual quarter milk samples which were obtained from 161 cows. Conventional antimicrobials including penicillin, kanamycin, gentamicin and erythromycin were used alone or combination for the treatment of clinical mastitis on these farms.

One hundred and twenty one *S. aureus* isolates from bovine milk were identified by biochemical methods, including a test for catalase, a tube coagulase test and anaerobic fermentation of mannitol. The identification was also confirmed by PCR amplification and sequencing of species-specific region of the gene encoding 16S rRNA and by amplification of thermonuclease gene (*nuc*) [10].

Antibiotic susceptibility testing was performed by the standard disc diffusion method on Mueller-Hinton agar (Oxoid, UK) plates according to Clinical and Laboratory Standards Institute (CLSI) guidelines [11, 12]. The antimicrobial agents (Oxoid, England) tested were (the disk concentration of the antibiotics indicated in parentheses): penicillin G (10 U), ampicillin (10 μg), gentamicin (10 μg), kanamycin (30 μg), tetracycline (30 μg), erythromycin (15 μg), clindamycin (2 μg) and chloramphenicol (30 μg) as in our previous studies [10, 13]. In addition, the susceptibility of antibiotics trimethoprim/sulfamethoxazole (SXT, 1.25/23.75, 25 μg) was conducted in the present study. *S. aureus* ATCC 25923 was used as a quality control. Results of susceptibility testing were recorded according to the CLSI [11, 12] guidelines following incubation at 35 °C for 18 h.

### Detection of three classes of integrases, gene cassettes and 3′ conserved region

Plasmid and chromosome DNA of bacterial isolates were extracted using a commercial DNAout kit (Tiandz Inc., Beijing, China) as described previously [13]. The presence of class 1, 2, and 3 integrons was analyzed in all isolates in the present study. The primers were used to detect the *intI*1 [6], *intI*2 [6] and *intI*3 [14] genes as in previous studies using a touchdown program: 94 °C for 5 min, ten touchdown cycles of 94 °C for 30 s, 68 °C for 30 s (decreasing by 1 °C /cycle) and 72 °C for 1 min, followed by 30 cycles of 30 s at 94 °C, 30 s at 58 °C and 1 min at 72 °C, with a final extension for 10 min at 72 °C (C1000™ Thermal Cycler, BIO-RAD, Beijing, China). The PCR products of the class 1 integrase gene (*intI*1) were randomly selected for sequencing to ensure specificity and accuracy. Amplicons of *intI*1 were purified with a PCR purification kit (TransGen Biotech, Beijing, China), ligated into the *pEASY*®-T5 Zero Cloning Vector (TransGen Biotech, Beijing, China), and then sequenced at Nanjing GenScript Ltd. (Nanjing, China). Sequence comparisons were performed using the BLAST program (http://www.ncbi.nlm.nih.gov/BLAST/). In addition, 2 × *TransTaq*®-T PCR SuperMix (+dye) (TransGen Biotech, Beijing, China) was employed to amplify the variable region (gene cassettes) between the *intI*1 gene and the 3’CS of class 1 integron using primers sets 5’CS and 3’CS as described previously [14] and following a three-step program [15] with minor modifications: 1 min of denaturation at 94 °C, 1 min of annealing at 52 °C and 2 min of extension at 72 °C for a total of 35 cycles; 5 s were added to the extension time at each cycle. Furthermore, two genes strongly associated with Class 1 integrons, the non-mobile cassettes conferring resistance to sulfadiazine, *sul*1, and to quaternary ammonium compounds, *qacΔE*-1, were also detected [6].

### Restriction fragment length polymorphism (RFLP) and DNA sequencing analysis of cassette regions

To determine whether each cassette gene PCR amplicon with the same size had the same content, cassette PCR products were digested with *Alu*I or *Rsa*I (New England Biolabs, Beijing, China). The PCR products with the same RFLP pattern were considered to contain same gene cassettes. To analyze the sequences of the gene cassettes, 1 or 2 cassette amplicons representative of each restriction profile were excised, purified and ligated into *pEASY*®-T5 Zero Cloning Vector (TransGen Biotech, Beijing, China). The ligation mixture was transformed into *Trans*-T1 Phage Resistant Chemically Competent Cell (TransGen Biotech, Beijing, China) and recombinants were selected on Luria agar containing ampicillin (100 mg/L). Recombinant plasmid DNA was purified by standard methods and subjected to sequencing at Nanjing GenScript Ltd. (Nanjing, China). The nucleotide sequences were analyzed and compared with available sequences in GenBank using the BLAST program by the National Center for Biotechnology Information (http://www.ncbi.nlm.nih.gov/blast/).

## Results

### Antimicrobial resistance profiles

High levels of resistance to antibiotics had been found in the bovine milk-associated *S. aureus* isolates, including to penicillin G (97/121, 80.2%), ampicillin (97/121, 80.2%), kanamycin (83/121, 68.6%), gentamicin (82/121, 67.8%), tetracycline (52/121, 43%), erythromycin (40/121, 33.1%; 38 isolates exhibited the inducible macrolide-lincosamide-streptogramin resistance phenotype and 2 isolates expressed the constitutive macrolide-lincosamide-streptogramin resistance phenotype and chloramphenicol (36/121, 29.8%) in our previous reports using the same isolates [10, 13]. Furthermore, 32.2% (39/121) of the isolates were resistant to SXT in the present study (Table 1). In addition, 89.3% (108/121) of the isolates were resistant to at least one antimicrobial, 47.1% (57/121) to three or more different classes of antimicrobials.

**Table 1:** Antimicrobial resistance phenotypes^a^ and genotypes

Antimicrobial subclass	Antimicrobial agent	Phenotypes		Genotypes (No. of isolates contained different resistance gene cassettes or type)				
		No. of resistant isolates (%)	No. of sensitive isolates	*aadA1, aadA2, aadA4, aadA5, aadA24, aacA4 or aadB*	*dhfrV, dfrA1* or *dfrA12*	*qacH*	*cmlA6*	type H integron only
Aminoglycosides	gentamicin	82(67.8)	–	61	/	/	/	21
		–	39	24	/	/	/	6
	kanamycin	83(68.6)	–	62	/	/	/	21
		–	38	25	/	/	/	6
Folate pathway inhibitors	trimethoprim/sulfamethoxazole (SXT)	39(32.2)	–	/	28	/	/	5
		–	82	/	46	/	/	20
Phenicols	chloramphenicol	36(29.8)	–	/	/	/	5	4
		–	85	/	/	/	13	23
Pencillins	Penicillin G	97(80.2)	/	/	/	/	/	25
		–	24	/	/	/	/	3
	ampicillin	97(80.2)	/	/	/	/	/	25
		–	24	/	/	/	/	3
Macrolides	erythromycin	40(33.1)	/	/	/	/	/	11
		–	81	/	/	/	/	16
Lincosamides	clindamycin	2(1.7)	/	/	/	/	/	1
		–	119	/	/	/	/	26
Tetracyclines	tetracyclin	52(43)	/	/	/	/	/	11
		–	69	/	/	/	/	12

^a^Part of phenotypes has been published in our previous studies [10, 13]

### Detection of class 1, 2 and 3 integrons

One hundred and twenty-one bovine milk associated *S. aureus* isolates were assayed by PCR amplification for the three classes of integrase genes. All isolates yielded a 280 bp PCR products, none of the isolates yielded 233 and 717 bp products. These suggested that all tested isolates had the class 1 integrase gene (*intI*1), with no class 2 and 3 integrase genes detected. Furthermore, all isolates were positive for the presence of *qacΔE-1*1 and *sul*1 genes, which are also the markers for class 1 integrons.

### Characterization of antibiotic resistance gene cassettes in class 1 integrons

Antibiotic resistance genes were detected using a CS-based PCR assay in which the primers flanked the variable region of the integron. Among 121 *intI*1 gene positive *S. aureus*, 85.1% (103/121) of isolates carried different gene cassettes.

**Fig. 1:**
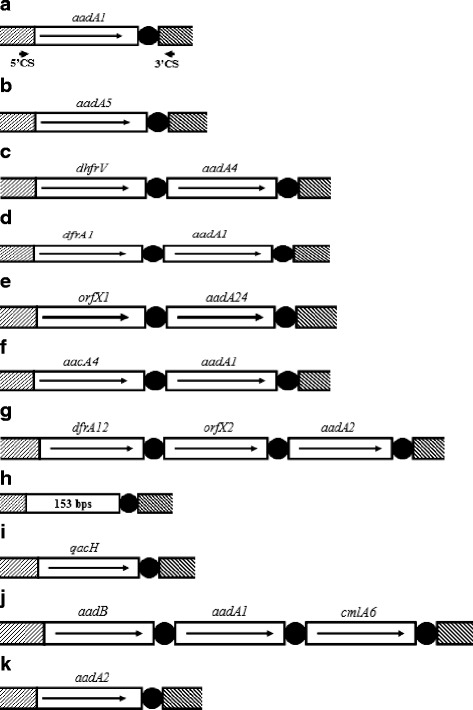
Schematic representation of the variable regions identified in bovine milk associated isolates. Cassette types (**a**-**k**) are arranged as identified in Table 2. Gene cassettes are shown as boxes, with arrows for the orientation of transcription and black circles for the 59-base elements. Hatched rectangles designate 5′- and 3′- conserved segments (CSs). The 5’CS and 3’CS amplification primers are indicated by arrowheads

Sequence analyses of PCR products representing each of the PCR-RFLP profiles identified a single gene cassette in the amplicons with sizes of 781 bp (A), 1048 bp (B), 874 bp (I) and 1009 bp (K). Two gene cassettes present in tandem were detected in the 1360 bp (C), 1586 bp (D), 1608 bp (E) and 1648 bp (F) amplicons, and three gene cassettes were present in the 1913 bp (G) and 3149 bp (J) amplicons. The 153 bp (H) amplicon was found to be a class 1 integron variable region without any gene cassette but contained partial sequences of 5′ and 3′ conserved segments of integrons. The resistance gene cassettes of those isolates are shown in Table 2, while the schematic representation of the inserted gene cassettes is shown in Fig. 1. In the present study, we found 2 open reading frames with unknown functions (*orfX1* and *orfX2*) and one 153 bp amplicon (H). The remaining genes encoded resistance to trimethoprim (*dhfrV, dfrA1* and *dfrA12*), aminoglycosides (*aadA1, aadA5, aadA4, aadA24, aacA4, aadA2* and *aadB*), chloramphenicol (*cmlA6*) and quaternary ammonium compound (*qacH*) (Table 2). Furthermore, 11 distinct kinds of gene cassette arrays (A-K) were identified (Table 2, Fig. 1). Of them, the gene cassette arrays *dfrA1-aadA1* (D, 44.6%), *aadA2* (K, 31.4%), *dfrA12-*hypothetical protein-*aadA2* (G, 27.3%) and *aadA1* (A, 25.6%) were found to be most prevalent. In addition, among 103 gene cassettes-positive isolates, 74 isolates contained 2 to 4 gene cassettes combinations, 26 isolates with H only and 3 isolates with G only (Table 3). The most frequent combinations were D + K + I + H (22 isolates) and J + G + D (18 isolates). To our knowledge, those gene cassette arrays have not been reported in bovine milk-associated *S. aureus* isolates previously, although all of these gene cassette arrays exist in other types of bacteria.

**Table 2:** Different gene cassette amplicon and numbers of isolates containing the gene cassette among 121 bovine milk associated *S. aureus*

Cassette types	Amplicon size (bp)	Resistance gene (s)	No. of isolates (%)
A	781	*aadA1*	31 (25.6)
B	1048	*aadA5*	3 (2.5)
C	1360	*dhfrV*	4 (3.3)
		*aadA4*	
D	1586	*dfrA1*	54 (44.6)
		*aadA1*	
E	1608	hypothetical protein (*orfX1*)	3 (2.5)
		*aadA24*	
F	1648	*aacA4*	4 (3.3)
		*aadA1*	
G	1913	*dfrA12*	33 (27.3)
		hypothetical protein (*orfX2*)	
		*aadA2*	
H	153	*–*	71 (58.7)
I	874	*qacH*	2 (1.65)
J	3149	*aadB*	18 (14.9)
		*aadA1*	
		*cmlA6*	
K	1009	*aadA2*	38 (31.4)

**Table 3:** Different combination of gene cassette arrays among 121 bovine milk associated *S. aureus* isolates

Gene cassette arrays	No. of isolates (%)
H	26 (21.5%)
D+ K + A + H	22 (18.2%)
J + G + D	18 (14.9%)
G + H	8 (6.6%)
D + K + H	8 (6.6%)
D + A	5 (4.1%)
F + C + B + A	3 (2.5%)
G	3 (2.5%)
E + K + H	2 (1.7%)
G + K + H	2 (1.7%)
G + K + I	2 (1.7%)
D + H	1 (0.83%)
F + C + H	1 (0.83%)
K + A	1 (0.83%)
G + E + H	1 (0.83%)

### The association between integron/cassettes and antimicrobial resistance

All of the integron/cassettes-positive isolates were resistant to aminoglycoside antibiotics. However, no gene cassette conferring resistance to erythromycin, clindamycin, tetracycline, penicillin and ampicillin was identified in the present study, thus the gene cassette arrays observed in our study did not account for those antimicrobial resistance phenotypes.

Among 39 SXT resistant and 36 chloramphenicol resistant *S. aureus* isolates, 28 and 5 of them contained gene cassettes *dhfrV, dfrA1* or *dfrA12* and *cmlA6,* 6 and 7 isolates were negative for any gene cassettes, 5 and 4 isolates were positive for the type H integron (153 bp), respectively. On the other hand, among 82 SXT susceptible and 85 chloramphenicol susceptible *S. aureus* isolates, 45 and 13 *S. aureus* isolates contained gene cassettes *dhfrV, dfrA1* or *dfrA12* and *cmlA6,* respectively. The rest of susceptible isolates harbored other cassettes (Table 1).

## Discussion

To the best of our knowledge, this is the first report of the presence of a class 1 integron and the resistance gene cassettes in bovine milk associated *S. aureus* isolates in China. The emergence of different resistance gene cassettes/arrays may reflect antibiotic selective pressure in our region, and provide useful antimicrobial surveillance information for the rational and effective use of antimicrobials. Different types of class 1 integrons were characterized which carried a variety of antibiotic resistance gene cassettes/arrays. All bovine milk-associated *S. aureus* isolates were carried by class 1 integrons. However, previous studies have documented a lower proportion of class 1 integrons among *S. aureus* isolates from hospital or farm animals, such as 53% (16/30) of clinical and environmental hospital-associated *S. aureus* isolates [7], 42.5% (76/179) of nosocomial methicillin-resistant *S. aureus* strains in China [8]. The differences between our *S. aureus* isolates and others’ reported ones may reflect the difference in selection pressure due to antibiotics use in our studied region. The class 1 integrons play a major role in the acquisition, expression, and dissemination of antibiotic resistance genes and may potentially have the capability to integrate nearly all the antimicrobial resistant genes such as β-lactamases, aminoglycosides, chloramphenicol, sulfonamides, macrolides, trimethoprim, fosfomycin, quinolone and quaternary ammonium compound [16] to disseminate multidrug resistance in clinical bacteria isolates. In our study, we found that aminoglycosides resistance determinants (*aadA1, aadA5, aadA4, aadA24, aacA4, aadA2* and *aadB*), trimethoprim resistance genes (*dhfrV, dfrA1* and *dfrA12*) and chloramphenicol resistance determinants (*cmlA6*) were prevalent among pathogenic *S. aureus* isolated from milk of mastitic cows. Those antimicrobial-resistance genes organized into 11 different gene cassettes arrangements (Fig. 1) and 74 isolates contained different combinations of gene cassette arrays, such as 22 isolates with the combination D+ K + A + H and 18 isolates with the combination J + G + D (Table 3). This may be due to the fact that aminoglycosides, chloramphenicol and trimethoprim were often widely used for the prevention or treatment of bovine mastitis in the past years in our region.

A BLAST search for our resistance gene cassettes in GenBank revealed high similarities were found with the published sequence in *Salmonella* or *Escherichia coli* isolates from different geographical locations or sources. This data suggests that class 1 integron/cassettes may contribute significantly to the horizontal transfer of antimicrobial resistance genes among different bacterial species from different sources or geographical locations [16]. However, the gene cassettes observed in class 1 integrons examined in our study did not account for the total resistance phenotypes observed (erythromycin, tetracyclines, clindamycin and pencillins antibiotics) among the pathogenic *S. aureus* isolates (Table 1). This suggests bovine milk associated *S. aureus* resistance to erythromycin, tetracyclines and pencillins may be not associated with integron-mediated antimicrobial resistance. And this could possibly reflect the presence of other mobile DNA elements, such as plasmid and transposon, or other resistance genes that we could not detected in this study.

The occurrence of resistance gene cassettes associated aminoglycosides resistance genes including *aadA1, aadA5, aadA4, aadA24, aacA4, aadA2* and *aadB* were relatively high among the *S. aureus* isolates. Similarly, a large proportion was reported in the case of clinical isolates of *Escherichia coli* in China hospital [14] and *Salmonella enterica* isolates from animal and human in Iran [4]. Furthermore, extensive existence of a variety of *aadA* alleles among *S. aureus* isolates indicates frequent recombination events in integrons. Similar allelic diversity has also been reported in *Escherichia coli* [17]. Therefore, class 1 integron/cassettes mediated capture of gene cassettes conferring resistance to aminoglycosides agents may play an important role among the antibiotic-resistant *S. aureus* isolates obtained from bovine milk. In addition, aminoglycoside antibiotic (neomycin, kanamycin or gentamicin) alone or combined with an antibiotic from another family (pencillins, ampicillin, macrolides or tetracyclines) has been widely used in the treatment of *S. aureus* intramammary infection on the local dairy farms. The class 1 integron exhibits a number of characteristics that might have adopted to antimicrobial agents by obtaining diverse gene cassettes with resistant genes. Human activities promote the diversification and dissemination of class 1 integrons [18].

In the present study, 45 and 13 isolates had the *dhfrV, dfrA1* or *dfrA12* and *cmlA6* gene cassettes while being phenotypically susceptible to SXT and chloramphenicol, respectively. The reasons for this phenomenon may be due to the presence of the weak promoter (strong promoter Pc variant) or repressors led to gene cassette expression transcriptional inactivation [19, 20]. The resistance gene cassette expression also can be affected by differences in the length and the sequence of *attC* sites, since the *attC* sites are imperfect inverted repeats that may form stem-loop structures similar to ρ-independent transcription terminators once they have been transcribed [19, 21]. From the clinical standpoint, those susceptible *S. aureus* isolates may be the genetic source for the evolution of resistance to clinically relevant antibiotics through mutation of regulatory sequences or integrase-mediated gene cassette rearrangements [19].

## Conclusions

In summary, all 121 tested bovine milk-associated *S. aureus* isolates carried class 1 integrons and 103 isolates contained different gene cassettes and we have presented evidence for the first time for class 1 integron as possible antibiotic resistance determinants on Chinese dairy farms.

## Abbreviations

3’CS: 3′ conserved segment
5’CS: 5′ conserved segment
*S. aureus*: *Staphylococcus aureus*
SXT: Trimethoprim/sulfamethoxazole
